# Quantitation and Identification of Therapeutic Anti-CD22 Monoclonal Antibodies in a Cell-Based ELISA Method

**DOI:** 10.3390/antib11030053

**Published:** 2022-08-16

**Authors:** Shengyu Fu, Qi Zhao

**Affiliations:** 1 Cancer Centre, Institute of Translational Medicine, Faculty of Health Sciences, University of Macau, Taipa, Macau SAR 999078, China; 2 MoE Frontiers Science Center for Precision Oncology, University of Macau, Taipa, Macau SAR 999078, China

**Keywords:** CD22, ELISA, monoclonal antibodies

## Abstract

Since they lack native soluble membrane antigens, the analysis and selection of antigen-specific antibodies are commonly performed on whole live cells. Here, we have developed a simple and convenient enzyme-linked immunosorbent assay (ELISA) based on cell membrane antigens. Soluble cell membrane proteins isolated from Raji cells were immobilized on the polystyrene microplate, which permitted the assessment of a therapeutic anti-CD22 monoclonal antibody. The experiments showed less variability in the intra-assay. Compared to the living cell ELISAs, the advantage of the assay is avoiding cell losses and high variation of optical density (OD) readings. We provide a quantitative and reproducible ELISA that can be potentially applied to the development of specific antibodies against cell surface antigens.

## 1. Introduction

Therapeutic monoclonal antibodies (mAbs) have become important for the treatment of diseases in clinics [1,2]. A standardized method is important for quality evaluation and pharmacokinetic (PK) measurements in preclinical and clinical trials of the administered mAbs [3]. Because soluble membrane antigens are not available or accessible, different alternative methods have been developed for the detection and analysis of mAbs that recognize cell surface antigens. For example, ELISA assays based on whole live cells [4,5,6] or fixed cells [6,7,8] have been used to characterize antibodies against cell surface antigens. Meanwhile, other methods based on anti-idiotype (anti-Id) antibodies [6,9], peptide conjugates [10], or flow cytometry [11] provide effective tools for monitoring the antibody production or PK of the administered antibody in the patients [12]. However, the development of anti-Id antibodies remains challenging, laborious, and time-consuming [13,14].

A number of anti-CD22 mAb drugs have been developed in different clinical trials for therapy of B-cell leukemias and autoimmune diseases [15,16]. Several anti-CD22 mAb are investigated under different clinical trial phases for non-Hodgkin’s lymphoma (NHL) [17,18,19]. CD22 is restrictively expressed in lymphocytes of the B cell lineage and can be found in the cytoplasm of pro- and pre-B cells. Because anti-CD22 mAbs can target and suppress matured B cells, their indications have been further expanded for the treatment of rheumatoid arthritis (RA), systemic lupus erythematous (SLE), etc. Therefore, it is urgently necessary to develop consistent and reliable protocols for quality control (QC) analysis of anti-CD22 mAbs and derivatives during antibody production or clinical studies [12,20].

Here, we showed a reliable and convenient ELISA assay based on whole cell membranes to assess anti-CD22 antibodies. Membrane proteins were isolated from Raji cells, a malignant B cell line, and then attached to polystyrene plate in carbonate-bicarbonate buffer. We assayed chimeric anti-CD22 antibodies to the membrane antigens of Raji cells. We further selected high-affinity phage-displayed binders from 11 anti-CD22 scFv candidates by using the established cell-based ELISA assay. This simple ELISA can be applied for anti-CD22 antibodies in QC analysis.

## 2. Materials and Methods

### 2.1. Antibodies and Cell Lines

Mouse/human chimeric anti-CD22 IgGs were generated by fusing the heavy and light chain sequences of the anti-CD22 antibody RFB4 with human IgG1 constant regions. The human B-lymphoblastoid cell line Raji was purchased from ATCC and cultured in RPMI 1640 supplemented with 10% fetal bovine serum (FBS) (GIBCO).

### 2.2. Expression and Purification of Anti-CD22 IgGs

The soluble anti-CD22 IgG protein was expressed by the HKE293T cells as per the procedures previously described [21,22,23].

### 2.3. Preparation of Cell Membrane Proteins

To prepare cell membrane antigen, 1 × 10^7^ Raji cells were harvested via centrifugation at 300× *g* for 5 min. They were washed with 1 mL of phosphate buffer (PBS) once and suspended in 0.5 mL of PBS. The suspended cells were ultrasonicated on ice for 45 s with 12 watts of power output and then centrifuged at 300× *g* for 10 min. The supernatant was collected and diluted in the final 0.5 mL of PBS. The protein concentration of the supernatant containing soluble membrane proteins was quantified using a BCA^TM^ Protein assay Kit (Pierce, Rockford, IL, USA). The concentration was set at 1–1.5 mg/mL.

### 2.4. Screening of Phage-Displayed Anti-CD22 scFvs

Phages were prepared as described previously [24]. To rescue the phage from the individual clones, replicas of the master plate were prepared by adding 20 μL of overnight culture from the master plate to 1 mL/well of 2×YT medium supplemented with 2% glucose and ampicillin in a fresh 96-well cluster plate. After incubation for ~1.5 h at 37 °C with shaking at 250 rpm, M13KO7 helper phage was added to each well in the master plate. The phage infection was performed by incubating for 1 h at 37 °C with shaking at 250 rpm. The infected bacterial cells were collected by centrifugation at 3000× *g* for 10 min at room temperature. The medium was then quickly discarded and cells in each well were resuspended in 2×YT medium containing ampicillin and kanamycin. The master plate was incubated overnight at 37 °C with shaking at 250 rpm. The next day, the culture plate was centrifuged at 3000× *g* for 10 min at room temperature and the phage supernatant was directly used for ELISA.

### 2.5. Cell-Based ELISA Assay

The concentration of membrane antigen was adjusted to 1 mg/mL in carbonate–bicarbonate buffer (15 mM Na_2_CO_3_, 35 mM NaHCO_3_, pH 9.6) and added to a 96-well polystyrene microplate plate (IWAKI, Tokyo, Japan) in amounts of 100 μL. The plate was incubated overnight at 4 °C to allow membrane antigen to attach to the plate bottom. To block non-specific binding sites on the plate, 200 μL of boric acid buffer (26 mM Na_2_B_4_O_7_, 100 mM H_3_BO_3_, 0.1% BSA, 100 mM NaCl, 3 mM KCl and 0.5% Tween-20, pH8.0) were added to each well. The plate was then incubated at 37 °C for 1 h. Serially diluted IgGs or phages in 100 μL of boric acid buffer were added to the plate and incubated at 37 °C for 1 h. To remove the unbound IgGs or phages, the plate was washed three times with 100 μL of boric acid buffer. Bound IgGs were detected by adding 100 μL of horseradish peroxidase (HRP)-conjugated goat anti-human Fc antibody (Jackson ImmunoResearch, West Grove, PA, USA). Bound phages were detected by mouse anti-M13KO7 antibody (GE Healthcare, Chicago, IL, USA). After 1 hour of incubation at 37 °C, the plate was washed as described as above. The substrate *o*-phenylenediamine (Sigma-Aldrich, St. Louis, MO, USA) was added, and plates were incubated until the appropriate color had developed. The reaction was stopped by adding 100 μL of 40% H_2_SO_4_. The absorbance (λ = 450 nm) was measured with a µQuant^®^ microplate reader (Bio-Tek Instruments Inc., Winooski, VT, USA).

### 2.6. Data Analysis

Data analysis was performed using GraphPad Prism 8 (GraphPad Software, San Diego, CA, USA). The OD readings of sample wells were normalized by subtracting blank reading from that of sample wells. The averages of duplicate well OD readings as *y*-values were plotted against the logarithm of concentration as *x*-values. The data were analyzed using the four-parameter curve-fitting equation explained in detail in the GraphPad Prism 4.0 Software.

## 3. Results

### 3.1. Validation for Cell-Based ELISA

In an attempt to roughly isolate membrane antigens, whole live cells were subjected to three simple steps, including ultrasonic process, low-speed centrifugation, and high-speed centrifugation. Ultrasonic process with 12 watts of power output for 45 s was effective enough to fragmentate the whole live cells. Via subsequently low-speed centrifugation, which may reduce unbroken cells or larger fragments, and high-speed centrifugation, which may sedimentate insoluble membrane antigen fragments, membrane antigens were isolated rapidly.

**Figure 1:**
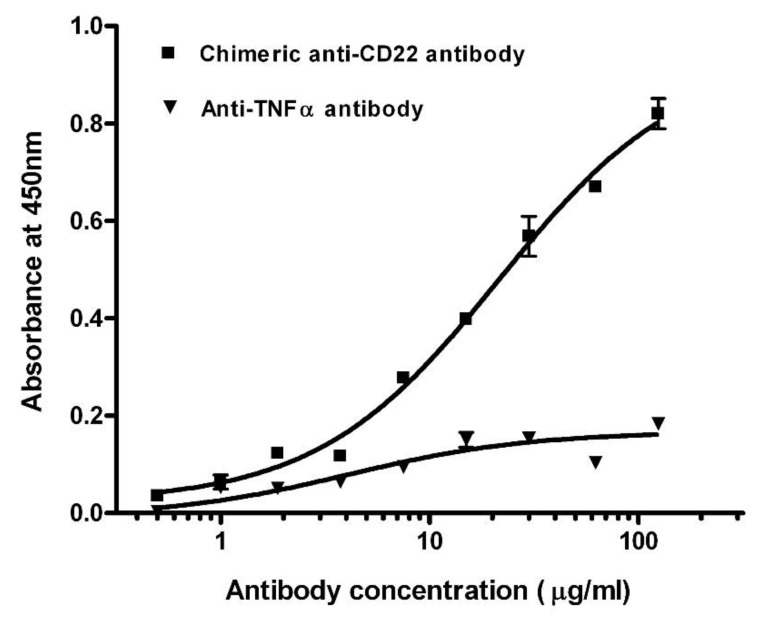
Titration curves of antibodies in binding assay against membrane antigens. Total soluble membrane proteins of Raji cells were used to detect chimeric anti-CD22 antibody (■) and anti-TNF α antibody (▼). The averages of samples were plotted against antibody concentration.

We screened the concentrations of membrane proteins at 0.5, 1, and 1.5 mg/mL in the preliminary experiments. After considering low non-specific background and high positive OD values, membrane proteins were adjusted to a final concentration of 1 mg/mL using carbonate–bicarbonate buffer (pH 9.6) and immobilized on the polystyrene microplate via overnight incubation at 4 °C. We assayed chimeric anti-CD22 antibodies against membrane antigens of Raji cells. Titration curves of the antibodies were plotted by the mean of OD reading against logarithm of concentration (Figure 1). The EC_50_ of chimeric anti-CD22 antibodies was shown as 28.3 μg/mL in the four-parameter curve-fitting analysis. EC_50_ may be used as an index of binding affinity of antibodies. To assess specificity of assay for measuring anti-CD22 antibodies, we used chimeric anti-CD22 antibodies as a positive control and an irrelevant anti-TNF alpha antibody to explore the antigen specificity of anti-CD22 antibody binding activity on membrane antigen of Raji cells. Chimeric anti-CD22 antibody displayed a dose response curve, whereas the irrelevant anti-TNF alpha antibody did not display detectable specific binding on membrane antigen of Raji cells. Results are shown in Figure 1. The results clearly prove the special binding of anti-CD22 antibodies to membrane antigen of Raji cells, whereas the irrelevant anti-TNF alpha antibody did not display detectable specific binding on membrane antigen of Raji cells.

The repeatability and reproducibility of the assay are acceptable. Our data are analyzed by one four-parameter curve-fitting equation. The data analyzed using the four-parameter logistic model had been applied in the immunoassays [8,25,26]. The chimeric anti-CD22 antibody was assayed three times on different days. The mean of OD readings was plotted against the logarithm of concentration for experiments performed on different days. Since EC_50_ may be used as an index of the binding affinity of antibodies, we analyzed the inter-assay and intra-assay EC_50_ values. The EC_50_ values of chimeric antibody both showed less variability in the inter- or intra-assay experiments. The EC_50_ values of chimeric antibody varied from 28.3 to 32.3, with an average of 29.5 ± 2.3 (CV = 10%), as shown in Table 1.

**Figure 2:**
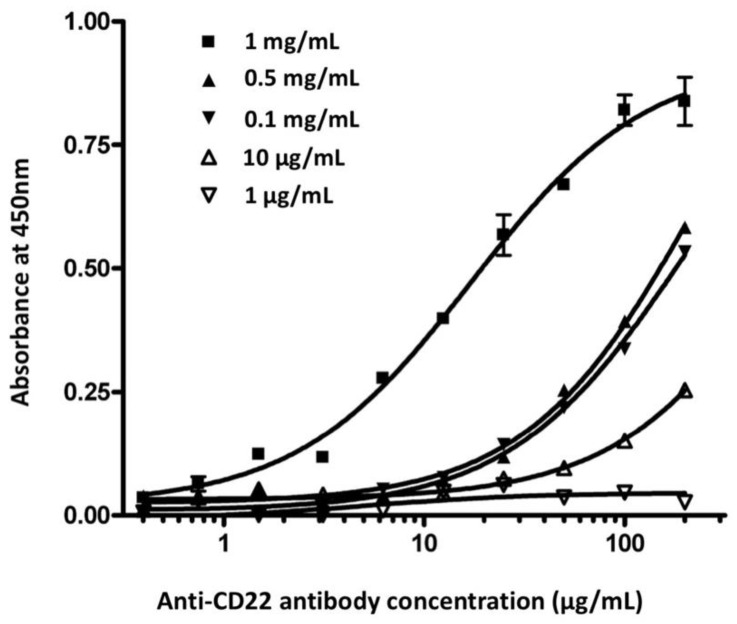
Effect of concentration of membrane antigen in chimeric anti-CD22 antibody binding assay. Membrane antigen was immobilized in a 96-well microplate at five different concentrations (1 μg/mL; 10 μg/mL; 0.1 mg/mL; 0.5 mg/mL; 1 mg/mL). Various concentrations of chimeric anti-CD22 antibody were used to detect membrane antigen. The averages of samples were plotted against the antibody concentration.

To investigate whether the concentration of the membrane antigen had an effect on the assay performance, we compared chimeric anti-CD22 antibody binding curves at five different concentrations of membrane antigens (Figure 2). All other assay conditions were the same for the 96-well plates. At the low 1 μg/mL and 10 μg/mL concentrations of membrane antigen, chimeric anti-CD22 antibody showed low binding affinity. The binding affinities of antibodies rose with the elevation of membrane antigen density. No significant difference in binding activity was observed at concentrations between 0.1 mg/mL and 0.5 mg/mL. The curve was obviously shifted at the concentration of 1 mg/mL. The optimal concentration of membrane antigen was determined to be 1 mg/mL. We coated 10 ng of recombinant CD22 protein per well in the ELISA plate. ELISA reached the saturation value at the 1 μg/mL concentration of anti-CD22 antibody. Cell-membrane-based ELISA reaches saturation at 100 μg/mL of anti-CD22 antibody. We estimate that the coated cell membrane contains CD22 amounts lower than 10 ng per well.

### 3.2. Selection of Binding-Improved Antibodies Using Cell-Based ELISA

**Figure 3:**
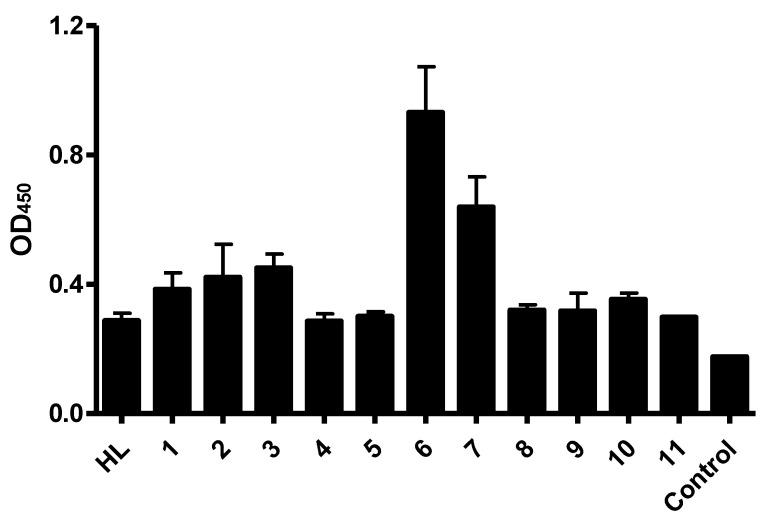
Binding characteristics of phage-displayed anti-CD22 scFvs. Different scFv mutants were displayed with phagemid vector. Overnight cultures of scFv-phages were incubated with Raji cell membrane, and bound scFv-phages were detected with an HRP-conjugated anti-M13 secondary antibody. HL represents the parental scFv clone. M13KO7 helper phages are used as the negative control. Data shown are means ± SEM of 2–3 separate experiments each performed in triplicate.

In order to improve the binding activity of anti-CD22 antibody, RFB4 was converted into single chain variable fragments (scFv). In previous studies, we randomly introduced mutated residues in the variable regions of heavy or light chains of RFB4 via error-prone PCR (non-published data). After cloning randomly mutated sequences into a phage displayed vector, the CD22-positive phages were selected against the recombinant CD22 antigens. After sequencing, eleven sequences with different residue mutations were used as examples in the ELISA. We attempted to select the best binders from 11 anti-CD22 scFv candidates by using the established cell-based ELISA assay. CD22-specific scFv clones were displayed using a phagemid vector. Phages displaying the scFv mutants, including parental scFv HL, were produced, and binding characteristics of the scFv-phages were compared by cell-based ELISA. Separate experiments were performed three times in triplicate sample wells. All scFv candidates gave specific binding in phage ELISA when compared to that of M13KO13 helper phage control (Figure 3). Among them, Clone 6 exhibited the strongest binding, with almost three-fold improvement in each experiment.

## 4. Discussion

The ELISA assay based on whole live cells is favorable due to the naïve molecular structure of antigens on the cell surface. In case of native soluble membrane antigens being expensive and not easily expressed for use in quality evaluation and pharmacokinetic (PK) measurements of the administered antibodies in preclinical and clinical trials, many alternative assay approaches have been developed to assess the membrane-specific antibodies. Meanwhile, the difference between batches of recombinant antigens can result in the high variations in the studies. The assay’s major limitation is high variation of OD reading and complicated wash procedures. Despite the adoption of anti-Id antibodies overcoming the difficulty, the production of anti-Id antibodies is always time-consuming. Therefore, we developed a quantitative cell membrane ELISA assay for assessment of membrane-specific antibodies. The ELISA assay was validated by measuring anti-CD22 antibodies against Raji cells. The results were quantitative and reproducible. When the method was further used to select CD22-scFv binders with improved binding in a phage manner, it exhibited reliable and significant results in three separate experiments. The ELISA method will be potentially useful in the high-throughput screening of antibodies.

Based upon these experiments, we demonstrate that the coating of membrane antigen to polystyrene plate in carbonate–bicarbonate buffer is tight enough so that the wash procedures can be performed on an ELISA microplate under vigorous conditions. Compared to the live cell ELISAs, the main advantage in the assay is avoiding cell loss and high variation of OD readings. This assay proves handy for evaluating antibodies targeting specific antigens on the cell surface.

## Figures and Tables

**Table 1:** Summary of intra-assay and inter-assay variability of EC50 of chimeric anti-CD22 antibodies.

	Experiment			Mean
	1	2	3	
EC50 (μg/mL)	28.3	28.09	32.27	29.55
SD	1.12	1.16	1.17	
SEM				2.35
CV%	3.9	4.1	3.6	7.9
*N* *	3	3	3	3

* Number of sample or experiment.

## Data Availability

All related data and methods are presented in this paper. Additional inquiries should be addressed to the corresponding author.
